# Host species identity, compartment, and site jointly shape the bryophyte-associated bacterial communities in subtropical area

**DOI:** 10.1186/s12866-026-05335-7

**Published:** 2026-06-26

**Authors:** Jin-Ye Liu, Yuan-Lan Ou-Yang, Jin-Hui Liu, Tao Wu, Cheng-Guang Xing, Chao-Jian Hu, Meng Ji, Wei-Qiu Liu

**Affiliations:** 1 Guangdong Provincial Key Laboratory of Plant Stress Biology, School of Ecology, Sun Yat-Sen University, Shenzhen, Guangdong 518107 China; 2 Jiangxi Guanshan National Nature Reserve, Yichun, Jiangxi 336300 China; 3 School of Life Sciences, Sun Yat-Sen University, Guangzhou, Guangdong 510275 China

**Keywords:** Bryophyte-associated bacteria, Compartment, 16S rRNA, Community assembly, Functional profiles, Co-occurrence network

## Abstract

Understanding the bacterial communities in non-vascular plants is essential for revealing the full landscape of plant–microbe interactions. However, comprehensive knowledge about bryophyte-associated bacterial communities remains limited, particularly in subtropical regions. Here, we investigated the bacterial communities of three compartments (soil, phyllosphere, and endosphere) from ten bryophyte species in two subtropical National Nature Reserves using 16S rRNA gene amplicon sequencing. We assessed how host species identity, compartment, and sampling site shape bacterial assembly, function and interactions. Our findings demonstrate that bacterial α diversity, community structure, and predicted functional profiles are influenced by the complex interplay among host species identity, compartment, and sampling site, whereas taxonomic composition and assembly processes are predominantly governed by the compartment. Compared to the soil, the phyllosphere and endosphere communities exhibit a higher proportion of stochastic processes as well as higher clustering coefficients and shorter average path lengths within the networks. Furthermore, taxa more abundant in the endosphere compared to soil exhibit compositional divergence and functional convergence across sites, characterized by higher proportions of terpenoid, polyketide, and xenobiotic metabolism pathways, alongside lower proportions of fundamental biosynthetic pathways. Overall, this study demonstrates that bryophyte-associated bacterial communities are jointly shaped by host species identity, compartment, and environment, and provides key insights into the ecological mechanisms of more extensive plant-microbe interaction patterns.

**Supplementary Information:**

The online version contains supplementary material available at https://doi.org/10.1186/s12866-026-05335-7.

## Introduction

Understanding the assembly, function, and interaction traits of plant-associated microbial communities is central to revealing plant-microbe interactions, predicting ecosystem functions and their responses to global change. Vascular plants (tracheophytes) and bryophytes are the two main lineages of extant land plants [1]. Although research on vascular plant associated microbiomes has systematically revealed their community formation and assembly traits, interaction patterns, and functional characteristics, the universality of these principles in bryophytes remains unknown [2–5]. Unlike vascular plants, bryophytes (include mosses, liverworts, and hornworts) are dominated by the gametophyte and without the lignified water-conducting cells. Despite their small size and simple structure, bryophytes comprise the high diversity with approximately 22,000 species [6], and colonize diverse habitats ranging from deserts to wetlands and from tropical to polar regions [7]. This adaptability may be not only attributed to their unique and divergent physiological adaptability, but also the close interaction relationship formed with microorganisms. A key evidence is that bryophytes possess a substantially greater diversity of gene families than vascular plants, with the acquisition from microbial genes proven to be a major driver of this diversity [8]. Given these distinct physiological and genomic characteristics, we hypothesize that assembly, function, and interaction traits of bryophyte-associated bacterial communities may be different with vascular plants.

Plant microbiome formation and assembly is a complex and non-random ecological process, influenced by complex interactions among environments (biotic or abiotic factors and different microbial pools), host compartment niches (different physicochemical properties, metabolism, immune response, and microhabitats), host identity (different species and genotypes), and microorganisms (competition, mutualism, predation, and trade-offs) [9, 10]. In vascular plants, the significant influence of compartment, host identity, developmental stage, soil management and seasonality on associated bacterial and fungal community assembly was proved in various experimental studies [11–18]. Although the relative predominance of stochastic and deterministic processes varies across studies, they revealed a shift toward greater importance of stochastic processes along the gradient from the rhizosphere to the phyllosphere, as environmental fluctuations intensify [19–21]. In contrast, researches on bryophyte-associated microbiomes exhibits significant limitations and imbalances, with disproportionately centered on the phyllosphere [22–24], with limited attention to endosphere [25] and soil microbial communities [26, 27]. Regarding microbial community assembly, research on *Sphagnum* has revealed deterministic processes as dominant drivers of methanogenic and methanotrophic community assembly [28], while a comprehensive understanding of assembly mechanisms underlying the entire bryophyte-associated microbiome remains elusive. Furthermore, most research has focused on alpine regions and on model taxa such as *Sphagnum* and *Physcomitrella*, leaving a notable gap in studies of highly biodiverse subtropical regions and other bryophyte lineages.

Understanding not only the assembly but also the interactions and functional capacities of bryophyte-associated microbiomes is essential for revealing their ecological roles. Microbial co-occurrence networks, widely used to infer interaction patterns and community stability, provide a powerful framework for this purpose. Studies of vascular plants show that microbial diversity and network complexity progressively decline from soil to the phyllosphere and further to the endosphere [17, 29]. Functionally, vascular plant microbes contribute broadly to host growth, reproduction, nutrient acquisition, disease resistance, and stress tolerance [10, 30]. Although studies describing interaction patterns among bryophyte-associated microbiomes are relatively scare, emerging research has increasingly revealed a broad spectrum of functional roles, including plant growth promotion, enhanced resilience to environmental stress, and contributions to biogeochemistry cycling, underscoring their ecological significance [23, 31–33]. Particularly, an increasing number of studies have focused on bryophyte-associated nitrogen-fixing microorganisms [34, 35]. Despite these advances, researches into the interaction patterns and functional differentiation of bryophyte-associated microbiomes across compartments remains limited, which constrains our understanding of how bryophyte–microbe symbiotics maintain functional efficiency and environmental adaptability.

To address these research gaps, this study systematically selected eight moss species and two liverwort species from the Guanshan (GS) and Chebaling (CBL) National Nature Reserves in the subtropical region of China, and investigated their associated bacterial communities across the endosphere, phyllosphere, and underlying soil. Using 16 S rRNA gene high-throughput sequencing, we established a multidimensional comparative framework that integrates host species identity, compartments, and sampling sites. Specifically, we addressed the following questions: (1) How do host species identity, compartment, and site shape the assembly of bryophyte-associated bacterial communities? (2) How do compartments and sites influence microbe-microbe interactions and keystone taxa? Through this work, we aim to establish a broader ecological framework for understanding the assembly and dynamics of microbiomes in non-vascular plants, while providing new insights into the evolutionary and adaptive mechanisms that underlie plant–microbe systems.

## Materials and methods

### Sample collection

**Fig. 1:**
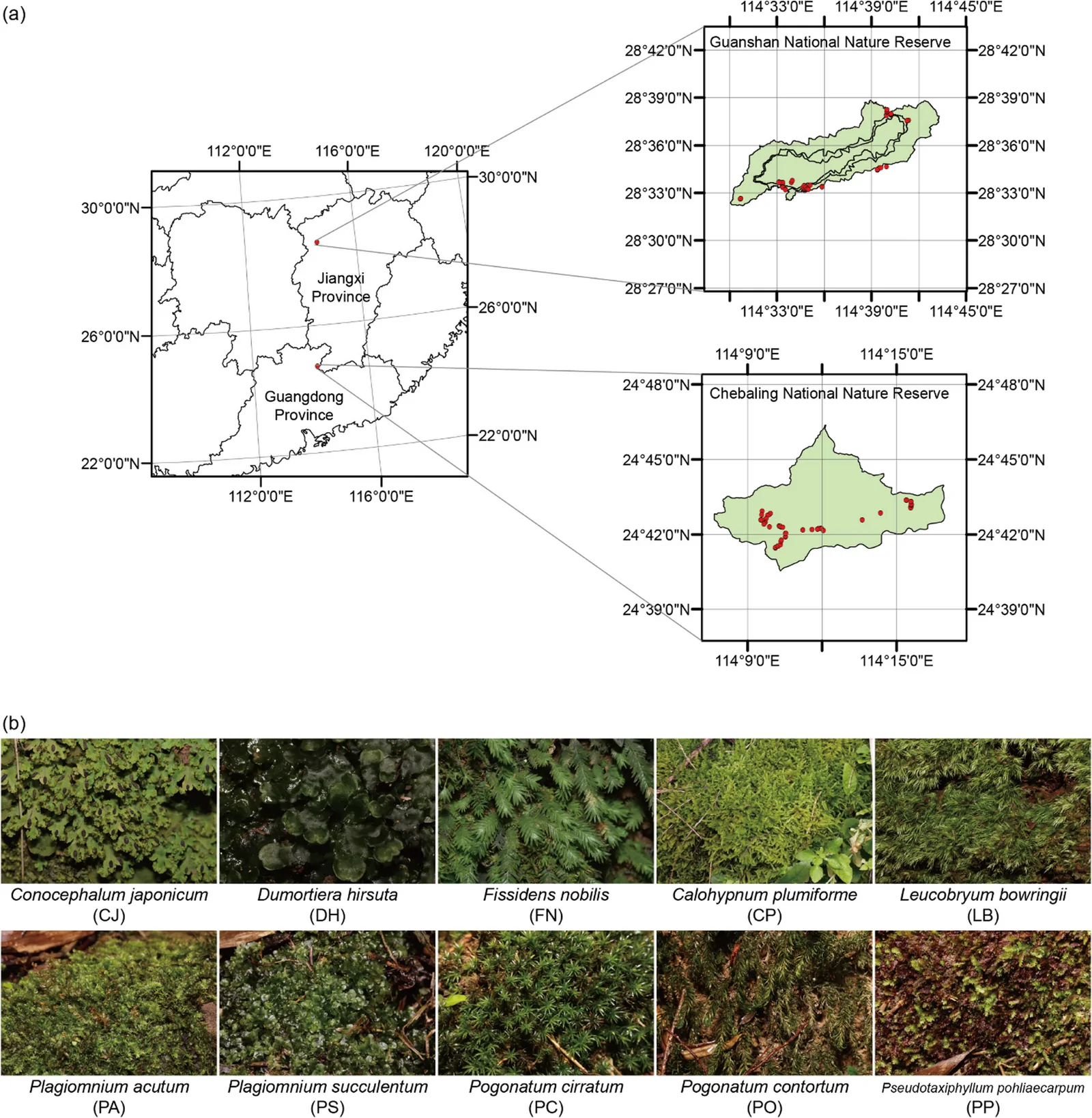
The information of sampling sites and host species in this study. **a** Geographic distribution of sampling spots in GS and CBL, indicated by red dots. **b** Photographs of the ten bryophyte species sampled. Abbreviations in parentheses denote species names, with CJ and DH representing Marchantiophyta (liverworts), and the remaining species belonging to Bryophyta (mosses)

Guanshan National Nature Reserve (GS; 114°29′E-114°45′E, 28°30′N-28°40′N) is situated in Jiangxi Province, China, with an annual average temperature of 16.2℃ and annual precipitation ranging from 1,950 to 2,100 mm. Chebaling National Nature Reserve (CBL; 114°09′E-114°16′E, 24°40′N-24°46′N) is located in Guangdong Province, China, characterized by an annual average temperature of 19.6℃ and annual precipitation of 1,468 mm (Fig. 1a). Both reserves are located in the subtropical areas.

Sampling of all bryophytes and associated soils was conducted with permission from the administrations of the Guanshan and Chebaling National Nature Reserves, and no special licenses were required. All sampled bryophyte species were morphologically identified by Weiqiu Liu, and voucher specimens have been deposited in the Museum of Biology, Sun Yat-sen University, with corresponding deposition numbers provided in Table S1.

Bryophyte samples (two liverworts and eight mosses) and the underlying topsoil beneath bryophyte mats (0–3 cm depth) were collected from GS and CBL in August–September 2021 (Fig. 1b). To minimize interference from other plant species, sampling spots were selected where only the target bryophyte species was present, and all spots were positioned at least 0.2 m away from adjacent shrubs and trees. Replicate samples were spaced at least 50 m apart. For each bryophyte species, 20 g of bryophyte material and 100 g of soil (remove stones, bryophyte rhizoids, and litter) were collected separately, with at least three replicates per species whenever possible. However, due to field constraints, four sampling groups (the phyllosphere of *Conocephalum japonicum* (CJ) in CBL, the phyllosphere of *Dumortiera hirsute* (DH) in GS, and the soil bacteria of *Plagiomnium succulentum* (PS) in both CBL and GS) had only two replicates.

Fresh bryophyte tissues (upper 2 cm of gametophytes) were rinsed with sterile water to remove surface dust. For phyllosphere DNA extraction, ~ 0.5 g of cleaned tissue was placed into sterilized bags. For endophytic DNA extraction, ~ 0.5 g of cleaned tissue was surface-sterilized in 75% (v/v) ethanol for 3 min, rinsed five times with sterile water, and transferred to sterilized bags. Additionally, ~ 0.5 g of soil was used for soil microbial DNA extraction. All plant and soil samples were stored at -20℃ as soon as possible until DNA extraction.

### DNA extraction and sequencing

Total DNA from phyllosphere, endosphere and soil microbes was extracted using the phenol-chloroform method (see Supporting Information for details). DNA concentrations were determined with a NanoDrop™2000 spectrophotometer (Thermo Fisher Scientific, USA). The V4 hypervariable region of the 16S rRNA gene was amplified with primers 515F (5’-GTGYCAGCMGCCGCGGTAA-3’) and 806R (5’-GGACTACHVGGGTWTCTAAT-3’), and sequencing was performed on the Illumina platform by Novogene Bioinformatic Technology Co., Ltd. (Tianjin, China). Raw reads were processed with QIIME2 [36], with low-quality sequences, barcodes and primers removed. And sequences were pair-end merged and quality-filtered (denoised, dereplicated, and chimera-filtered) using the DADA2 plugin [37]. Amplicon sequence variants (ASVs) were clustered with a 100% similarity threshold based on the SILVA_138 database [38]. Sequences assigned to chloroplasts and mitochondria were excluded from further analyses.

### Soil properties analysis

Soil pH was measured using an electronic pH meter (soil: water = 1:1, m/v). Soil organic matter (SOM) was determined via the potassium dichromate oxidation method. Total nitrogen (TN) content was quantified using the Kjeldahl digestion method [39]. Total phosphorus (TP) content was measured by the vanadium-molybdenum blue colorimetric method. Available nitrogen (AN) content was determined via the alkaline-hydrolysis diffusion method [40]. Available phosphorus (AP) content was analyzed using the Bray-1 Method.

### Statistics analysis

The Pearson correlation was used to assess the relationships between soil properties and the relative abundances of the top 10 soil bacterial classes in GS and CBL. ASVs with total abundance < 10 across all samples were removed, and the remaining ASVs were rarefied to the minimum read count (21,056 reads) for diversity analyses. Alpha diversity (Richness, Simpson, Shannon) was calculated using the *vegan* package in R [41], and differences across host species identity, compartments, and sampling sites were tested using Wilcoxon or Kruskal–Wallis tests. A generalized linear model (GLM) was used to examine the main and interaction effects of host species identity, compartment, and sampling site on richness. Community structure and functional structure were visualized by Principal Coordinate Analysis (PCoA) based on Bray–Curtis dissimilarity, and the effects of host species identity, compartment, and sampling site were assessed via Permutational Multivariate Analysis of Variance (PERMANOVA) using the *adonis* function in R package *vegan* [41]. Functional profiles of bacterial communities were predicted using PICRUSt2 with KEGG pathway annotation [42, 43].

The relative contributions of deterministic and stochastic processes in bacterial community assembly were evaluated using the beta Nearest Taxon Index (βNTI) and the Bray–Curtis-based Raup-Crick index (RC_Bray_) using the *picante* package in R [44–47]. The main effects of host species identity and compartment on these processes were examined using GLM. Distances in taxonomic composition and predicted functional profiles of bacterial communities were illustrated using hierarchical agglomerative clustering with Ward’s minimum variance method based on Bray–Curtis distances [48].

Significantly enriched or depleted ASVs in endophytes relative to soil bacteria was identified using the GLM approach of edgeR [49], excluding ASVs with a total abundance ≤ 50 across all samples. Enriched or depleted ASVs were defined as those with |log₂ fold change| > 2 and *P*_adjusted_ < 0.05. Significant differences in KEGG level two metabolic pathways of enriched ASVs between endophytic and soil bacterial communities were assessed using Welch’s *t*-test in STAMP [50] (*p* < 0.05).

Bacterial co-occurrence networks at the ASV level were inferred using SPIEC-EASI with the Meinschausen-Buhlmann method (nlambda = 20, lambda.min.ratio = 0.01), and the optimal model was selected via StARS (rep.num = 50, thresh = 0.05) using the *SpiecEasi* package in R (https://github.com/zdk123/SpiecEasi) and visualized with Gephi [51–53]. For each network, ASVs with a total relative abundance greater than 0.01% and present in at least 20% of the samples were retained. Network modular structure was identified using the Louvain algorithm. Topological properties were calculated using the *igraph* package in R [54]. Nodes were determined based on Within-module connectivity (*Z*_*i*_) and participation coefficient (*P*_*i*_) following Guimerà and Amaral [55]. Nodes were classified as keystone taxa (network hubs (*Z*_*i*_≥2.5, *P*_*i*_≥0.62), module hubs (*Z*_*i*_≥2.5, *P*_*i*_<0.62), and connectors (*Z*_*i*_<2.5, *P*_*i*_≥0.62)), or peripherals (*Z*_*i*_<2.5, *P*_*i*_<0.62) [56, 57].

## Results

### Assembly of bacterial communities among host species identity, compartments, and sampling sites

**Fig. 2:**
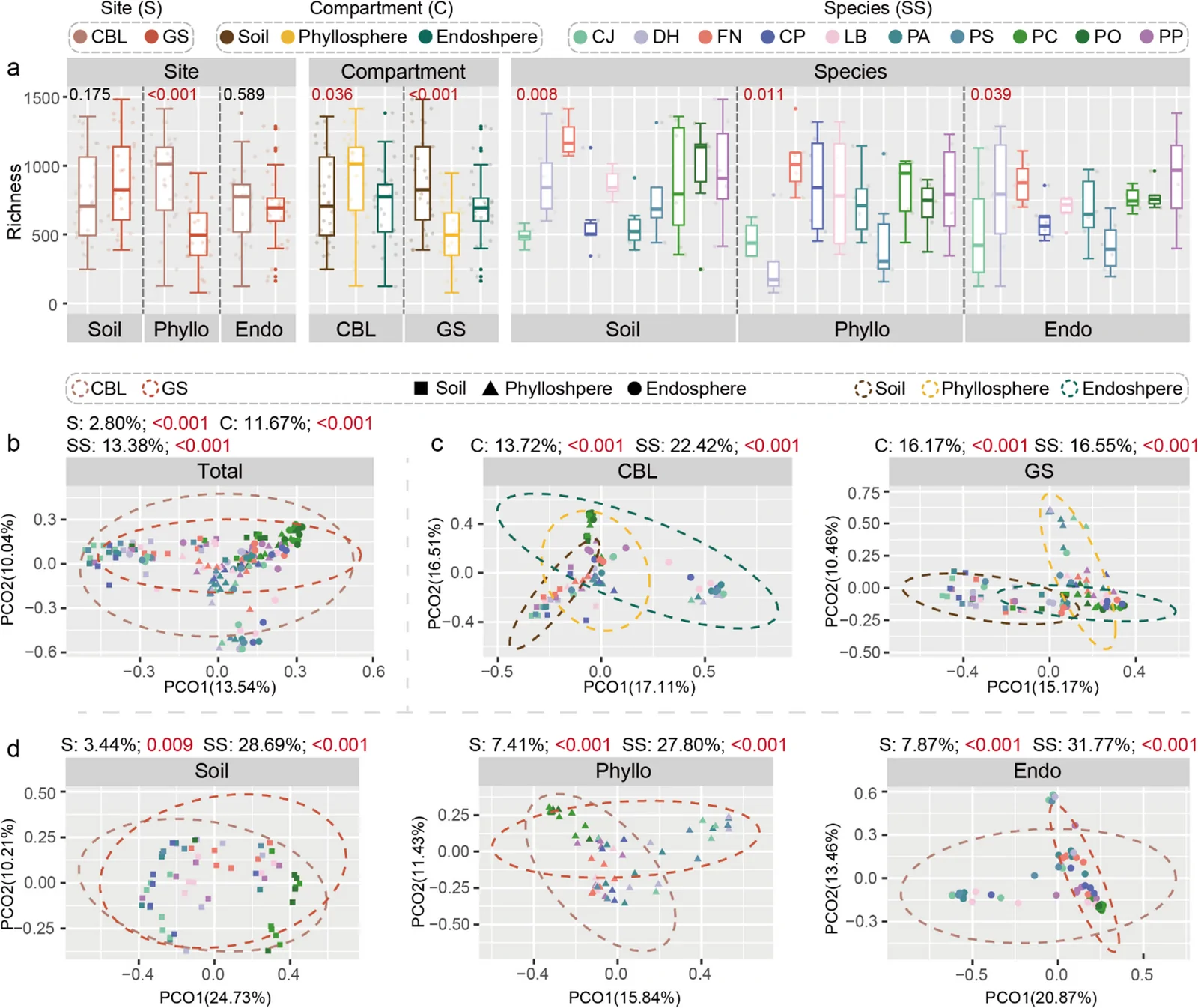
Bacterial alpha diversity and community structure across host species identity, compartments and sampling sites. **a** Richness in different host species, compartments and sampling sites. Numbers indicate *p* values from nonparametric Wilcoxon or Kruskal-Wallis tests. **b**-**d** Principal coordinate analysis (PCoA) plots based on Bray-Curtis dissimilarities of (**b**) all samples, (**c**) each sampling site, and (**d**) each compartment. The relative contribution and significance of different factors on community dissimilarity were assessed by PERMANOVA. ‘S’ denotes sampling sites, ‘C’ denotes compartments, and ‘SS’ denotes host species identity. Numbers above the figures (b-d) represent *R*^*2*^ and *p* values from PERANOVA, with *p* < 0.05 highlighted in red. ‘Phyllo’ indicates phyllosphere, and ‘Endo’ indicates endosphere

Our results demonstrate that the α diversity, community structure, and functional profiles of bryophyte-associated bacteria are driven by a complex interplay of host species identity, compartment, and sampling site, rather than a single universally dominant variable. For α diversity, GLM analyses revealed that the interaction between sampling site and compartment had the strongest effect (*p* < 0.001; Fig. 2a, Fig. S1a, Table S2). In addition, both the main effect of species (*p* < 0.001) and the interaction between compartment and species (*p* < 0.05) significantly influenced α diversity. Meanwhile, site (*p* = 0.015) and compartment (*p* = 0.022) also had significant effects on richness, which is more sensitive to rare taxa. For bacterial community structure, PERMANOVA further revealed that the strong main effects of host species identity (R^2^ = 13.38%), compartment (R^2^ = 11.67%) and their strong interaction (R^2^ = 12.60%, *p* < 0.001 for all), while the sampling site effect was minor (R^2^ = 2.80%, *p* < 0.001; Fig. 2b-d; Table S2). Similarly, predicted functional profiles were predominantly driven by complex interactions. The compartment × species (R^2^ = 14.31%, *p* < 0.001) and three-way interactions (R^2^ = 10.13%, *p* = 0.031) explained substantially more variation than the individual main effects of species, site, and compartment (all R^2^ < 6.00%, *p* < 0.050; Fig. S1b-d; Table S2).

Regarding taxonomic composition, while compartment was the primary driver for most dominant bacterial classes, interaction effects also played a crucial role, with their relative importance varying across classes (Fig. S2). Specifically, regarding the most abundant classes, soil communities were dominated by Bacilli and the phyllosphere by Gammaproteobacteria. In contrast, the endosphere exhibited a clear interactive pattern, being dominated by Gammaproteobacteria in CBL and Alphaproteobacteria in GS (Fig. S3). And pearson correlation analysis indicated that soil pH (< 7 in all samples, Table S3) significantly correlated with the relative abundance of dominant soil bacteria, showing positive correlations with Bacilli and negative correlations with several other classes in both CBL and GS (Fig. S4).

**Fig. 3:**
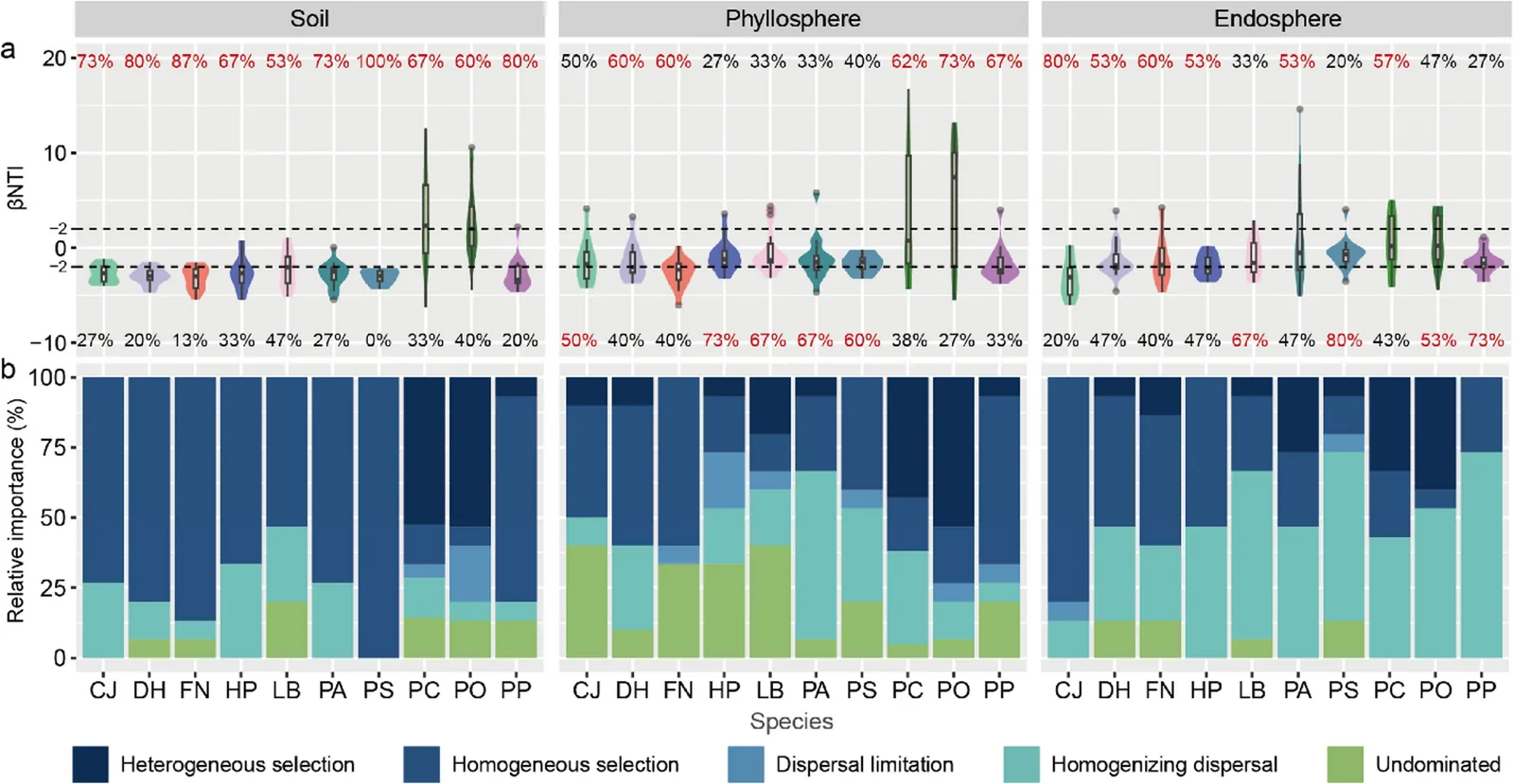
Deterministic and stochastic processes in bacterial community assembly across host species and compartments. **a** Relative contributions of deterministic (|βNTI| > 2) and stochastic (|βNTI| ≤ 2) processes, with the numbers above and below each violin plot denoting the proportions of deterministic and stochastic processes, respectively. Values exceeding 50% are highlighted in red. **b** Relative importance of five ecological processes based on βNTI and RC_Bray_ values. Heterogeneous selection and homogeneous selection are deterministic processes, while dispersal limitation, homogenizing dispersal, and undominated processes are stochastic processes

GLM analysis further indicated that compartment significantly shaped the relative contributions of deterministic and stochastic processes (*F* = 4.511, *p* = 0.016), whereas neither host species identity (*F* = 1.503, *p* = 0.175) nor site (*F* = 3.699, *p* = 0.061) had a significant effect. After merging data from the two sampling sites, null model analysis revealed distinct assembly processes across compartments (*F* = 7.871, *p* = 0.004). Specifically, deterministic processes dominated all soil bacterial communities (53–100%, 10 in 10), part phyllosphere (27–73%, 5 in 10) and endosphere communities (20–80%, 6 in 10; Fig. 3a, b).

The relative contributions of the five ecological assembly processes varied significantly across compartment and host species. Regarding deterministic processes, homogeneous selection was significantly influenced by both compartment (*F* = 7.756, *p* = 0.004) and host species identity (*F* = 3.090, *p* = 0.020). This process not only accounted for an extremely high proportion in soil communities but also emerged as the dominant deterministic process in most phyllosphere and endosphere communities. However, *Pogonatum cirratum* and *P. contortum* exhibited a distinct assembly pattern. Heterogeneous selection, strongly driven by host identity (*F* = 14.572, *p* < 0.001), accounted for a notably high proportion across all compartments in both species, whereas this process was almost absent in most other samples (Fig. 3a, b). Hierarchical agglomerative clustering based on ASV composition and predicted functional profiles showed that *P. cirratum* and *P. contortum* tended to cluster together in all three compartments at each site, indicating high similarity in both taxonomy and functional potential (Fig. S5).

Regarding stochastic processes, homogenizing dispersal was the predominant driving processes in endosphere communities. Its relative contribution was significantly shaped by compartment (*F* = 10.130, *p* = 0.001), and remaining relatively low in other compartments. Meanwhile, undominated processes were also significantly driven by compartment (*F* = 8.162, *p* = 0.003) and exhibited a relatively higher proportion in phyllosphere communities. Finally, dispersal limitation remained consistently low across almost all groups and showed no significant association with the examined factors.

### The composition and function of taxa more abundant in phyllosphere and endosphere compared to soil bacteria

**Fig. 4:**
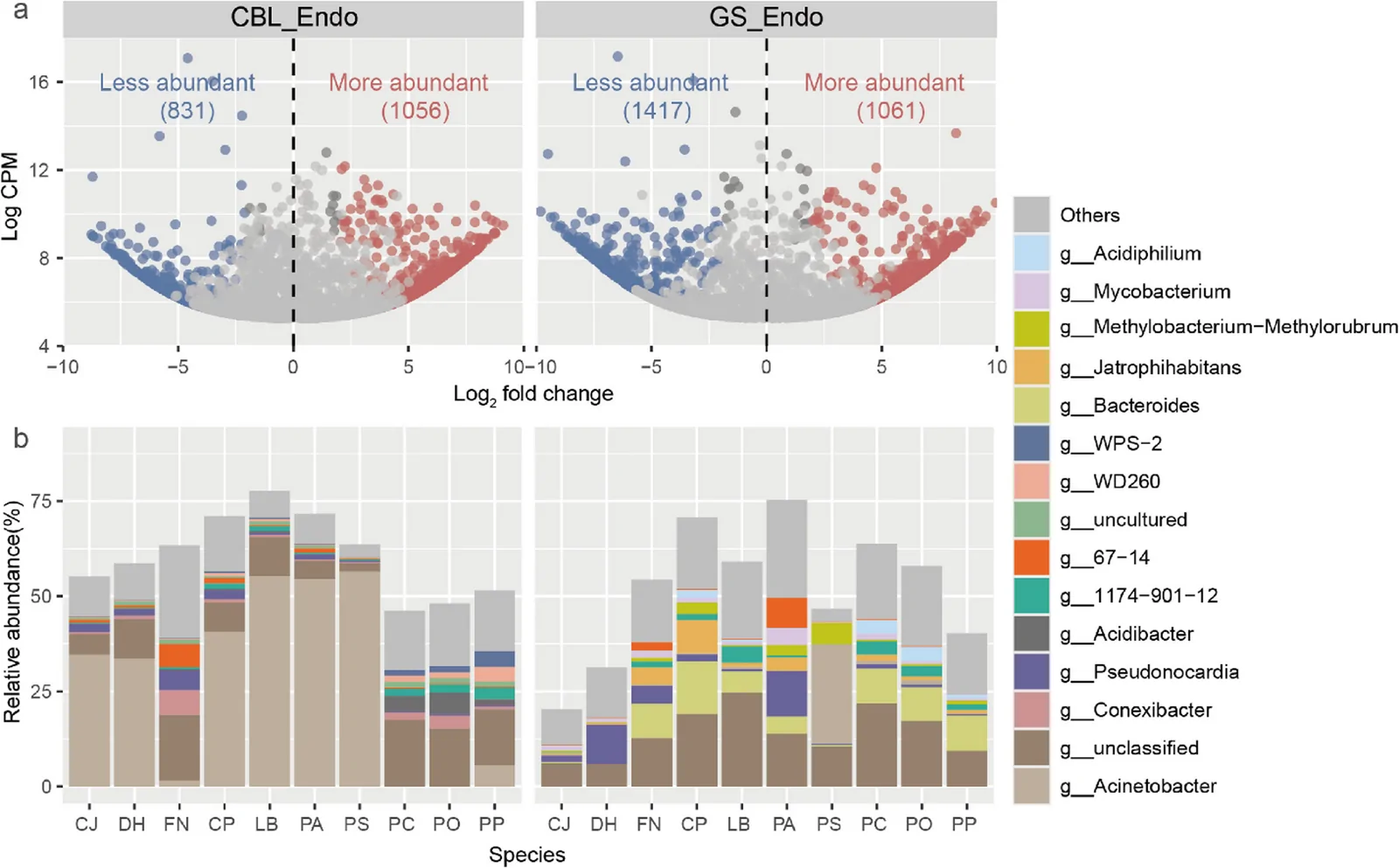
Numbers and taxonomic composition of ASVs more abundant in endophytic communities relative to soil bacterial communities at each sampling site. **a** Numbers of differentially abundant ASVs in endophytes relative to soil in CBL and GS. Red and blue dots represent the more and less abundant ASVs, respectively, with counts indicated. **b** Relative abundance of and taxonomic composition of the top 10 genera based on ASVs more abundant in CBL and GS

Endophytic bacteria, but not phyllosphere communities, exhibited a pattern of taxonomic divergence and convergent functional potential across sampling sites. For the endosphere, ASVs significantly more abundant compared to soil were comparable in number but distinct in composition between the two sites (Fig. 4a, b). At CBL, the 1,056 (25.93%) significantly more abundant ASVs were dominated by *Acinetobacter* in most samples, followed by unclassified genera, *Conexibacter*, *Pseudonocardia*, and *Acidibacter* (Fig. 4a, b). At GS, the 1,061 (22.74%) significantly more abundant ASVs were primarily composed of unclassified genera in most samples, followed by *Bacteroides*, *Pseudonocardia*, *Acinetobacter*, and *Jatrophihabitans* (Fig. 4a, b).

**Fig. 5:**
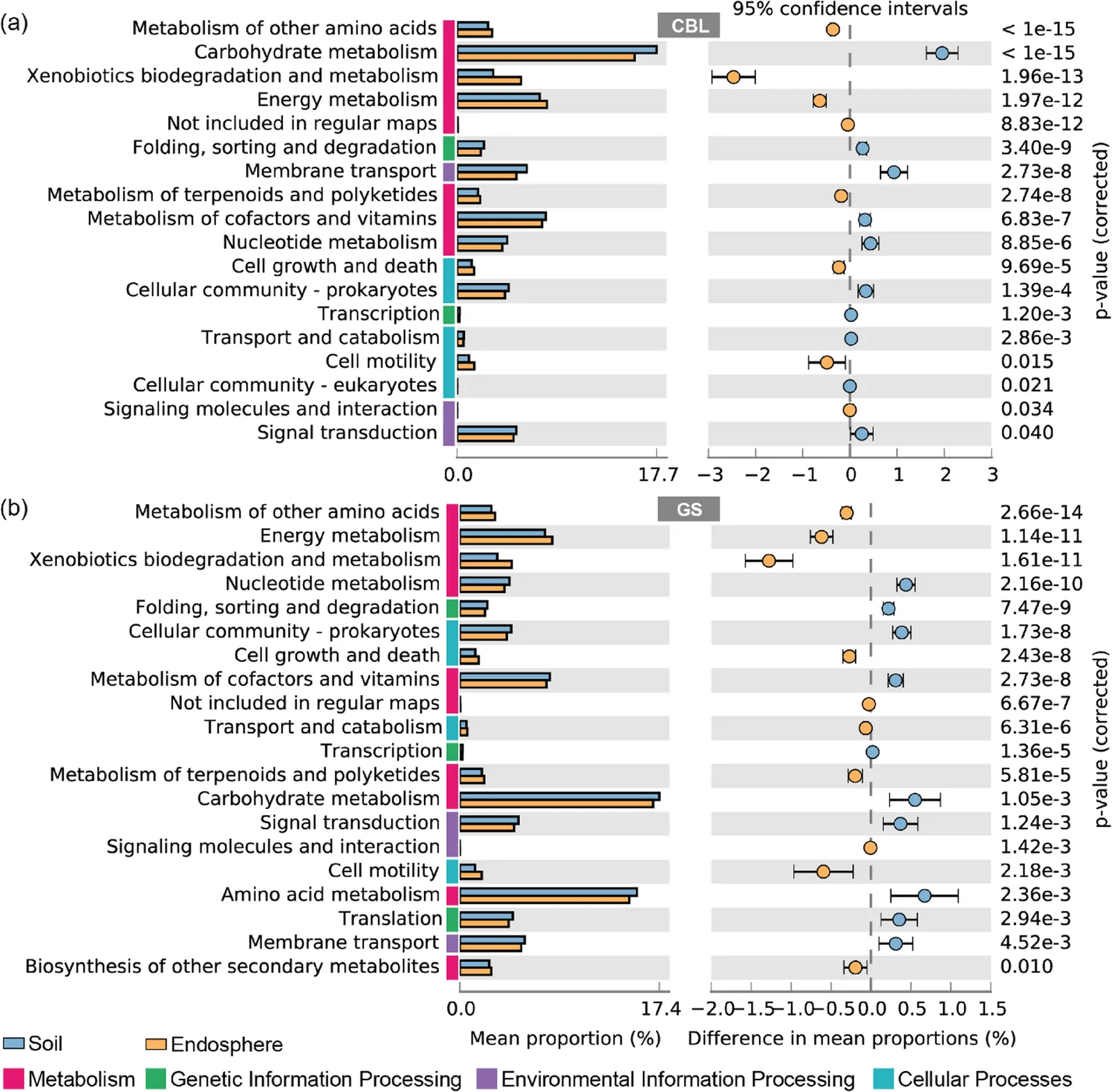
Significantly differences in KEGG level two metabolic pathways of differentially abundant ASVs between endosphere and soil bacterial communities in (**a**) CBL and (**b**) GS, assessed using Welch’s t-test

Despite the taxonomic divergence between sites, the predicted functional profiles of these endophytes showed strong convergence. Analysis of KEGG level 2 pathways revealed similar functional differences between these differentially abundant endophytic and soil communities at both CBL and GS (Fig. 5a, b). Pathways exhibiting significantly increased proportions in endophytes included metabolism of other amino acids, xenobiotics biodegradation and metabolism, energy metabolism, metabolism of terpenoids and polyketides, cell growth and death, and cell motility. In contrast, pathways showing significantly decreased proportions in endophytes related to soil communities included carbohydrate metabolism, folding, sorting and degradation, membrane transport, metabolism of cofactors and vitamins, nucleotide metabolism, cellular community – prokaryotes, and signal transduction.

A parallel analysis of phyllosphere communities revealed a different pattern. The taxonomic composition of ASVs significantly more abundant in the phyllosphere mirrored the site-specific patterns observed in the endosphere, with *Acinetobacter*-affiliated ASVs dominating in most CBL samples and unclassified genera prevailing in GS samples (Fig. S6). In contrast, the differences in predicted KEGG level 2 pathways between differentially abundant phyllosphere and soil bacterial communities were inconsistent between CBL and GS, indicating weaker functional convergence (Fig. S7).

### Co-occurrence network patterns and keystone taxa in different compartments

**Fig. 6:**
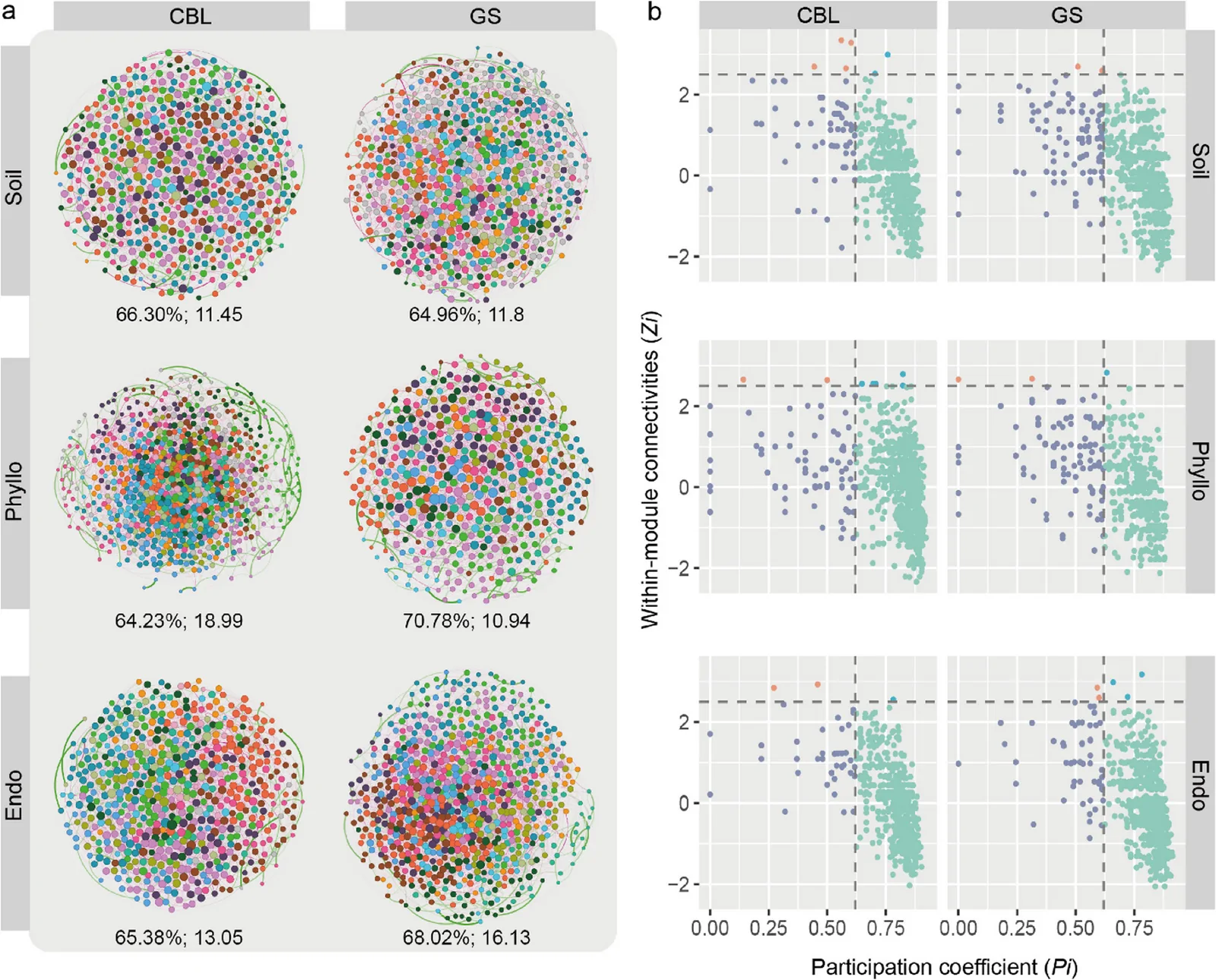
Co-occurrence network patterns of bacterial communities in different compartments and sampling sites. **a** The co-occurrence network of different bacterial communities. Green and red lines represent positive and negative correlations, respectively. Node size indicates the degree of ASVs, and node color indicates different modules. Numbers below the networks show the proportion of positive correlations and the average degree. **b** *Zi*-*Pi* plots showing the distribution of ASVs based on their topological roles in different networks. Nodes were classified as keystone taxa, (network hubs (*Z*_*i*_≥2.5, *P*_*i*_≥0.62), module hubs (*Z*_*i*_≥2.5, *P*_*i*_<0.62), and connectors (*Z*_*i*_<2.5, *P*_*i*_≥0.62)), or peripherals (*Z*_*i*_<2.5, *P*_*i*_<0.62)

To characterize bacterial interactions, ASV-level co-occurrence networks were constructed for the soil, phyllosphere, and endosphere communities at both sites (Fig. 6a). Overall, these networks were dominated by positive correlations (64.23%–70.78%) and exhibited great stability (0.040–0.050), high average degrees (10.94–18.99), and clear modularity (modularity: 0.43–0.55; modules: 14–21). Across compartments, phyllosphere and endosphere networks displayed higher clustering coefficients and lower average path lengths compared to the soil networks.

Keystone taxa varied markedly in proportion and taxonomy (Fig. 6b; Table S5). Connectors were highly dominant in both ASV count (76.18%–92.09%) and relative abundance (47.35%–66.61%), whereas module hubs and network hubs were exceedingly rare (2–4 and 0–5 ASVs with < 1% abundance). Taxonomically, connectors mainly comprised unclassified taxa and ubiquitous genera (e.g., *Acinetobacter*, *Acidibacter*, *Conexibacter*, and *Burkholderia-Caballeronia-Paraburkholderia*), with *Pseudomonas* and *Pseudonocardia* showing higher proportions in the CBL regions and endosphere, respectively. Conversely, both module hubs and network hubs were highly group-specific. Module hubs comprised unclassified taxa (in CBL soil, GS soil, and GS endosphere) and distinct genera (CBL soil: *Chitinophaga*; CBL phyllosphere: *Brevundimonas*; GS endosphere: *Conexibacter*; CBL soil: *Catenulispora*; GS phyllosphere: Muribaculaceae; GS endosphere: *Jatrophihabitans*). Network hubs similarly featured unique profiles of known genera (CBL soil: *Luedemannella*; CBL phyllosphere: *Acidothermus*, *Roseiarcus*, *Sphingomonas*; GS endosphere: B12-WMSP1, *Tumebacillus*; CBL endosphere: *Dyella*, *Methylobacterium-Methylorubrum*) alongside unclassified taxa in CBL phyllosphere, GS phyllosphere, and GS soil.

## Discussion

### Bryophyte bacterial communities are jointly shaped by host species identity, compartment, and sampling site

Similar to vascular plants [17, 22, 58, 59], bryophyte-associated bacterial communities were governed by the integrated effects of host species identity, compartment, and environment. Specifically, while bacterial α diversity is primarily governed by the interaction between site and compartment, taxonomic composition is dominantly driven by the compartment. And community structure is predominantly shaped by host species, compartment, and their interplay. In addition, predicted functional profiles depend on the compartment × species and overall three-way interactions. Fundamentally, these complex patterns reflect the holistic adaptation of microbiomes to morphological and physiological differences among host species, the unique microhabitats and metabolic signatures of different compartments, and the heterogeneous microbial source pools and physicochemical conditions across spatial sites [60]. For instance, pH was identified as a core soil factor significantly influencing the relative abundance of dominant soil bacteria, a finding that aligns closely with previous observations in moss-dominated peatland ecosystems [61].

Deterministic processes emphasized the drive of biotic and abiotic factors (e.g., host filtering, environmental conditions, and biotic interactions) for community structure. When these conditions are homogeneous, little variation in community structure or species/compositional turnover is expected, referred to as homogeneous selection [62]. In contrast, if environmental conditions change across space or time, high variation in community structure could exist, referred to as heterogeneous selection [63]. Stochastic processes emphasized the drive of birth, death, immigration and emigration, spatiotemporal variation, or historical contingency (e.g., colonization order) to community structure. Within a large region, degrees of dispersal could vary substantially among taxa [64]. High dispersal rates homogenize community structure, lead to little variation or turnover, referred to as homogenizing dispersal [46]. Low dispersal rates, coupled with drift or weak selection, increase community variation or turnover, often referred to as dispersal limitation [46]. In addition, other processes influencing community structure (e.g., diversification, ecological drift, historical contingency, and contemporary selection) are encompassed within undominated processes.

In this study, dispersal limitation was extremely low across all groups. This is likely due to the fact that both sampled reserves are subtropical evergreen broad-leaved forests with close proximity and similar climates. These factors collectively contributed to a relatively consistent microbial source pool across spatial scales. This also explains the high proportions of homogenizing dispersal and homogeneous selection across compartments. Under these similar regional conditions, the assembly of bryophyte-associated microbial communities is jointly driven by local selection pressures and stochastic disturbance. Similar to previous studies on vascular plants [19, 21], phyllosphere and endosphere microbiomes experiencing more intense environmental fluctuations exhibited the stronger stochastic processes. The significant increase of undominated processes in phyllosphere may also suggest that these fluctuations create more opportunity for stochastic events such as ecological drift [65]. However, distinct from other bryophyte species, heterogeneous selection replaced homogeneous selection as the dominant deterministic process in the bacterial communities of *P. cirratum* and *P. contortum*. This is likely due to their unique morphological and anatomical traits. Both of them are relatively tall and erect with conducting-like tissues [66]. These structures may exert a stronger shaping effect on local microenvironments, driving community heterogeneity at larger spatial scales while maintaining compositional and functional similarities locally. Taken together, these findings highlight the joint influence of sampling site, compartment, and host species in driving the assembly of bryophyte-associated microbiomes. Overall, these findings highlight the complexity of bryophyte-associated bacteria construction, provide a refined understanding of bryophyte–microbe interactions.

### Endophytic bacteria exhibit composition divergence and functional convergence across sites

The endophytic bacterial communities of bryophytes exhibited the pattern of taxonomic divergence but functional convergence, reflecting the strong and conserved functional filtering imposed by the host on its endophytic microbiota. Compared with soil microbes, the more abundant endophytic taxa across distinct sampling sites exhibit a consistently higher proportion of pathways linked to terpenoid and polyketide metabolism. This pattern aligns well with the known characteristics of bryophytes, which typically synthesize these metabolites [67, 68], and may suggests that endophytic bacteria are strongly adapted to the unique chemical environment within the host. Meanwhile, the decreased proportion of fundamental biosynthetic pathways, such as nucleotide, carbohydrate, cofactor, and vitamin metabolism, may indicate that endophytes increasingly depend on host-derived nutritional resources, thereby reducing their fundamental metabolic investment and achieving energy conservation and metabolic reconfiguration. Additionally, the increased proportion of xenobiotic biodegradation and metabolism pathways in endophytic microbes may imply an enhanced capacity of bryophytes to degrade environmental pollutants, cause *Sphagnum* has been demonstrated to adsorb and uptake functionalized nanoplastics [69]. Collectively, these compositional differences and functional parallels indicate that host selection may prioritize functional traits over taxonomic identity, while endophytes appear to utilize host resources and reinforce specialized functions that align with the host niche [70], thereby contributing to the stabilization of host–microbe interactions. This feature maybe widely prevalent in nature. For instance, the genetic and functional similarity exhibited by bacterial communities associated with diverse vascular plants [71], endophytic functions that facilitate self-colonization and promote host development [70, 72], and the reduction of metabolic diversity by microbes to adapt to the environment coupled with ubiquitous metabolic exchanges [73, 74], all further underscore the complex functional interactions between microorganisms, their hosts, and environments.

In contrast to endophytic bacteria protected by host tissue barriers, phyllosphere bacterial communities exhibit a more variable taxonomic composition and relatively weaker functional convergence across distinct sampling sites. This pattern suggests that, as the critical interface between the external environment and the host interior, the phyllosphere microbiota responds more directly to local microclimatic fluctuations and environmental stress than hosts [30]. Such characteristics enable the phyllosphere community to function as a sensitive indicator of environmental change, and also offer the host a reservoir of diverse microbial traits that can be recruited or filtered under changing conditions.

### Network and keystone taxa vary across compartments and sampling sites

Instead of traditional correlation-based methods (e.g., Spearman or Pearson), we employed the SPIEC-EASI approach, which is more robust for microbial interaction analysis. Our networks revealed consistently complex, stable, and modular structures characterized by high positive correlations and connectivity across sites and compartments. The dominance of positive correlations suggests that mutualistic cooperation prevails over competitive exclusion within these communities based on the stress-gradient hypothesis [75, 76]. Furthermore, the high connectivity and large proportion of connectors reflect highly complex interactions among microorganisms. Notably, the phyllosphere and endosphere networks exhibited higher clustering coefficients and lower average path lengths compared to soil, which may suggest that they maintain tighter interactions and efficient connectivity under fluctuating or stressful environmental conditions [77].

In these co-occurrence networks, connectors maintaining inter-module connectivity exhibited similarities across groups, whereas module hubs sustaining intra-module stability and network hubs integrating both roles displayed stronger compartment specialization. Together, they contribute a complex yet stable network. Although, keystone taxa varied across compartments and sites, many possess plant-beneficial functions. For example, genera such as *Acinetobacter*, *Pseudomonas*, *Pseudonocardia*, and *Sphingomonas* are capable of phosphorus solubilization or indole-3-acetic acid (IAA) production [78–81]; while *Burkholderia-Caballeronia-Paraburkholderia*, *Roseiarcus*, and *Methylobacterium-Methylorubrum* are associated with nitrogen fixation [82–85]. Additionally, study in boreal forests highlight the roles of moss-associated microbes in carbon fixation, nitrogen cycling, and methane metabolism, supporting tight moss-microbiome interactions [22]. Overall, for bryophytes lacking vascular systems and roots, this highly interconnected and functionally cooperative microbial network likely serves as a key compensatory mechanism facilitating nutrient acquisition and growth. These network-based structural and functional insights provide a novel perspective for understanding the dynamics of bryophyte-microbiome relationships.

## Conclusion

Our study provides the first investigation of bryophyte-associated bacteria across compartments in a subtropical region. The results indicate that bacterial α diversity, community structure, and predicted functional profiles are influenced by the complex interplay among host species identity, compartment, and site, whereas taxonomic composition and assembly processes are predominantly governed by the compartment. Compared to the soil, the phyllosphere and endosphere communities exhibit a higher proportion of stochastic processes as well as higher clustering coefficients and shorter average path lengths within the networks. Although bacterial compositional differences were observed between sites, taxa more abundant in the endosphere compared to maintained largely convergent functional profiles, and numerous keystone taxa were associated with plant growth promotion and stress tolerance. These findings offer novel insights into the assembly mechanisms and functional potential of bryophyte-associated microbial communities, highlighting their ecological adaptability and the role of host specificity in maintaining microbiome function across contrasting environments. It should be noted that this study was limited to sampling during summer at two reserves, and relied on 16 S rRNA-based predictions without experimental validation. Future studies incorporating multi-season sampling, multi-omics approaches, and physiological validation will help further elucidate how bryophyte-microbe interactions respond to environmental variation.

## Supplementary Information

Supplementary Material 1.

## Data Availability

All raw sequencing data have been submitted to the NCBI Sequence Read Archive (SRA) database under the accession number PRJNA1334295.
