# Supplementary material for: Host species identity, compartment, and site jointly shape the bryophyte-associated bacterial communities in subtropical area
Source: BMC Microbiol. 2026 Jun 26;26:795. doi: 10.1186/s12866-026-05335-7 (PMC13560375; doi:10.1186/s12866-026-05335-7)
Supplement: Supplementary file 1 [file 12866_2026_5335_MOESM1_ESM.docx]

**Host Species Identity, Compartment, and Site Jointly Shape the Bryophyte-Associated Bacterial Communities in Subtropical Area**

*Jin-Ye Liu^#^, Yuan-Lan Ou-Yang^#^, Jin-Hui Liu, Tao Wu, Cheng-Guang Xing, Chao-Jian Hu, Meng Ji and Wei-Qiu Liu^*^*

**Supplementary data**

**Detailed DNA extraction method**

A 0.5 g sample of treated bryophyte or soil was added to a mortar, followed by the addition of liquid nitrogen to grind the sample into a powder. The powder was transferred to a clean, sterile 1.5 mL centrifuge tube, and 460 μL of 1× TE buffer was added. Then, 40 μL of lysozyme, 50 μL of 20% SDS, and 8 μL of 20 mg/mL proteinase K were added, and the mixture was incubated overnight at 37°C in a shaker to release the DNA by disrupting the cytoderm and nucleoproteins. Next, 550 μL of phenol-chloroform-isoamyl alcohol (25:24:1) solution was added, and the mixture was vortexed until the biphasic layer disappeared, further denaturing proteins, inhibiting DNase degradation, and separating the DNA from proteins and other organic materials. Centrifuge the tube at 2,000×g for 10 minutes at room temperature to separate the phases. Transfer the upper aqueous phase to a clean, sterile 1.5 mL centrifuge tube. Add an equal volume of isopropanol and incubate at –20°C for 30 minutes. Centrifuge the tube at 12,000×g for 5 minutes and decant the supernatant. Add 1 mL of ethyl alcohol and incubate at room temperature for 1 minute. Centrifuge the tube at 12,000×g for 5 minutes, decant the supernatant, and invert the 1.5 mL centrifuge tube onto a paper towel for 5 minutes. Add 100 μL of 1× TE buffer to resuspend the DNA. DNA concentration was measured using the NanoDrop™2000 spectrophotometer.

**Supplementary table legends**

**Table S1:** Voucher specimen numbers of ten bryophyte species from GS and CBL National Nature Reserves

**Table S2:** Effects of sampling site, compartment, host species identity, and their interactions on bryophyte-associated bacterial α-diversity, community structure, and functional structure, as assessed using generalized linear model (GLM)

**Table S3:** Physicochemical properties of the topsoil beneath bryophyte mats in different sampling sites

**Table S4:** Topological properties of bacterial co-occurrence networks in different sampling sites and compartments

**Table S5:** Genus-level classification of keystone ASVs in bacterial co-occurrence networks of different sampling sites and compartments

| Bryophyte species | GS National Nature Reserve | CBL National Nature Reserve |
| --- | --- | --- |
| *Conocephalum japonicum* | gw2021084, gw2021104, gw2021116 | cw2021027, cw2021028, cw2021029 |
| *Dumortiera hirsuta* | gw2021010, gw2021039, gw2021050 | cw2021005, cw2021006, cw2021009 |
| *Fissidens nobilis* | gw2021041, gw2021065, gw2021069 | cw2021032, cw2021037, cw2021043 |
| *Calohypnum plumaeforme* | gw2021083, gw2021092, gw2021098 | cw2021013, cw2021014, cw2021042 |
| *Leucobryum bowringii* | gw2021017, gw2021048, gw2021080 | cw2021003, cw2021018, cw2021033 |
| *Plagiomnium acutum* | gw2021038, gw2021100, gw2021105 | cw2021015, cw2021031, cw2021041 |
| *Plagiomnium succulentum* | gw2021066, gw2021071, gw2021075 | cw2021034, cw2021039, cw2021040, |
| *Pogonatum cirratum* | gw2021023, gw2021054, gw2021061 | cw2021004, cw2021011, cw2021045 |
| *Pogonatum contortum* | gw2021020, gw2021046, gw2021081 | cw2021001, cw2021044, cw2021049 |
| *Pseudotaxiphyllum pohliaecarpum* | gw2021087, gw2021101, gw2021108 | cw2021002, cw2021007, cw2021020 |

**Table S1:** Voucher specimen numbers of ten bryophyte species from GS and CBL National Nature Reserves

|  |  | Site | Compartment | Species | Site:Compartment | Site:Species | Compartment:Species | Site:Compartment:Species |
| --- | --- | --- | --- | --- | --- | --- | --- | --- |
|  | df | 1 | 2 | 9 | 2 | 9 | 18 | 18 |
| Richness | *F* value | 6.137 | 3.934 | 7.501 | 16.971 | 1.925 | 1.886 | 0.774 |
|  | *p* | 0.015 | 0.022 | <0.001 | <0.001 | 0.055 | 0.023 | 0.727 |
| Shannon | *F* value | 0.011 | 0.297 | 14.570 | 7.975 | 1.024 | 2.071 | 1.219 |
|  | *p* | 0.917 | 0.743 | <0.001 | <0.001 | 0.425 | 0.011 | 0.257 |
| Simpson | *F* value | 1.742 | 1.165 | 13.123 | 6.750 | 1.040 | 2.508 | 1.349 |
|  | *p* | 0.189 | 0.316 | <0.001 | 0.002 | 0.412 | 0.002 | 0.171 |
| Community structure | *R^2^* | 2.80% | 11.70% | 13.40% | 2.90% | 5.50% | 12.60% | 8.00% |
|  | *p* | <0.001 | <0.001 | <0.001 | <0.001 | <0.001 | <0.001 | 0.002 |
| Functional structure | *R^2^* | 5.50% | 3.34% | 5.64% | 5.51% | 7.19% | 14.31% | 10.13% |
|  | *p* | <0.001 | 0.001 | 0.031 | <0.001 | 0.002 | <0.001 | 0.031 |

**Table S2:** Effects of sampling site, compartment, host species identity, and their interactions on bryophyte-associated bacterial α-diversity, community structure, and functional structure, as assessed using generalized linear model (GLM)

Values with *p* < 0.05 are highlighted in red.

**Table S3:** Physicochemical properties of the topsoil beneath bryophyte mats in different sampling sites

| Sites | Type | CJ | DH | FN | CP | LB | PA | PS | PC | PO | PP |
| --- | --- | --- | --- | --- | --- | --- | --- | --- | --- | --- | --- |
| CBL | pH | 5.58±0.26 | 5.8±0.8 | 5.29±0.56 | 5.35±0.38 | 4.3±0.39 | 5.78±0.84 | 5.86±0.69 | 4.65±0.17 | 4.71±0.17 | 4.57±0.05 |
|  | SOM | 33.25±9.77 | 30.85±20.1 | 39.67±24.79 | 41.36±6.03 | 77.01±46.59 | 45.3±10.72 | 164.16±126.14 | 35.19±13.94 | 31.71±17.56 | 38.63±9.39 |
|  | TN | 1.68±0.37 | 1.47±0.95 | 1.99±1.09 | 1.99±0.31 | 2.49±1.1 | 2.23±0.55 | 5.76±3.14 | 1.61±0.45 | 1.65±0.77 | 1.74±0.35 |
|  | TP | 0.56±0.07 | 0.56±0.44 | 0.49±0.15 | 0.37±0.18 | 0.46±0.25 | 0.45±0.21 | 0.38±0.08 | 0.31±0.07 | 0.68±0.46 | 0.42±0.09 |
|  | AN | 139.44±42.27 | 130.37±100.22 | 168.34±74.12 | 157.92±33.56 | 176.4±71.19 | 168±46.83 | 433.44±192.45 | 134.82±25.49 | 140.45±61.7 | 139.44±30.79 |
|  | AP | 2.2±0.14 | 1.51±1.11 | 1.29±1.04 | 13.08±9.85 | 1.63±1.19 | 8.64±6.49 | 22.19±26.46 | 1.47±0.16 | 1.7±1.22 | 1.67±0.87 |
| GS | pH | 5.96±0.06 | 5.08±0.11 | 5.04±0.16 | 5.7±0.08 | 4.73±0.16 | 5.49±0.49 | 5.13±0.39 | 4.7±0.13 | 4.49±0.18 | 4.8±0.28 |
|  | SOM | 25.35±25.47 | 95.7±34.29 | 62.42±19.96 | 38.41±11.46 | 115.29±71.26 | 220.22±271.52 | 190.31±110.25 | 41.32±19.05 | 93.51±37.89 | 58.99±33.8 |
|  | TN | 1.35±1.22 | 4.78±1.69 | 3.41±0.88 | 1.93±0.43 | 4.37±2.21 | 9.14±11.18 | 9.89±6.08 | 2.25±0.79 | 3.66±1.15 | 2.65±1.34 |
|  | TP | 0.35±0.02 | 0.85±0.17 | 0.69±0.14 | 0.36±0.05 | 0.48±0.22 | 1.07±0.81 | 1.46±0.79 | 0.45±0.16 | 0.48±0.2 | 0.44±0.11 |
|  | AN | 114.92±109.76 | 354.48±152.65 | 302.4±85.98 | 158.93±45.15 | 325.92±111.15 | 688.8±776.79 | 738.36±317.18 | 194.88±85.28 | 288.96±90.77 | 198.91±80.74 |
|  | AP | 8.08±5.56 | 18.17±16.21 | 8.26±5.13 | 4.45±2.62 | 4.42±2.8 | 86.83±110.94 | 51.88±45.64 | 1.45±1.2 | 4.98±1.74 | 3.76±2.09 |

SOM, soil organic matter; TN, total nitrogen; TP, total phosphorus; AN, available nitrogen; AP, available phosphorus.

SOM, TN, and TP: g/kg; AN, and AP: mg/kg.

**Table S4:** Topological properties of bacterial co-occurrence networks in different sampling sites and compartments

| Sites | Compartments | Number of nodes | Number of edges | Proportion of  positive correlation | Average degree | Average path length | Network diameter | Network density | Clustering  coefficient | Modularity |
| --- | --- | --- | --- | --- | --- | --- | --- | --- | --- | --- |
| CBL | Soil | 556 | 3184 | 0.66 | 11.45 | 2.92 | 5 | 0.02 | 0.07 | 0.50 |
|  | Phyllosphere | 785 | 7453 | 0.64 | 18.99 | 2.71 | 6 | 0.02 | 0.09 | 0.43 |
|  | Endosphere | 583 | 3804 | 0.65 | 13.05 | 2.84 | 5 | 0.02 | 0.08 | 0.48 |
| GS | Soil | 704 | 4152 | 0.65 | 11.80 | 2.98 | 5 | 0.02 | 0.06 | 0.48 |
|  | Phyllosphere | 466 | 2550 | 0.71 | 10.94 | 2.94 | 6 | 0.02 | 0.09 | 0.55 |
|  | Endosphere | 729 | 5881 | 0.68 | 16.13 | 2.78 | 5 | 0.02 | 0.09 | 0.46 |

**Table S5:** Genus-level classification of module hubs and network hubs in bacterial co-occurrence networks of different sampling sites and compartments

| Sites | Compartments | Keystone taxa | ASVs (Genus) |
| --- | --- | --- | --- |
| CBL | Soil | Module hubs | ASV115 (g__Chitinophaga); ASV643 (f__Vibrionaceae) |
|  |  | Network hubs | ASV1396 (g__Luedemannella) |
|  | Phyllosphere | Module hubs | ASV258 (g__Brevundimonas); ASV1734 (g__Geobacillus) |
|  |  | Network hubs | ASV141 (g__Sphingomonas); ASV525 (f__Solirubrobacteraceae); ASV828 (f__Xanthobacteraceae); ASV973 (g__Acidothermus); ASV1026 (g__Roseiarcus) |
|  | Endosphere | Module hubs | ASV135 (g__Conexibacter); ASV712 (g__WPS-2); ASV861 (g__67-14); ASV1549 (g__Nordella) |
|  |  | Network hubs | ASV44 (g__Methylobacterium-Methylorubrum); ASV228 (g__Dyella) |
| GS | Soil | Module hubs | ASV567 (c__Acidimicrobiia); ASV1030 (g__Catenulispora) |
|  |  | Network hubs | ASV113 (f__Solirubrobacteraceae); ASV1018 (g__B12-WMSP1); ASV2378 (g__Tumebacillus) |
|  | Phyllosphere | Module hubs | ASV405 (g__Muribaculaceae); ASV1683 (g__CL500-29_marine_group) |
|  |  | Network hubs | ASV198 (c__Acidimicrobiia) |
|  | Endosphere | Module hubs | ASV3728 (p__Patescibacteria); ASV520 (f__Microbacteriaceae); ASV851 (g__Jatrophihabitans) |
|  |  | Network hubs | - |

**Supplementary figure legends**

**Figure S1:** Bacterial alpha diversity (a) and functional structure (b-d) across host species identity, compartments and sampling sites. (a) Shannon and Simpson in different host species, compartments and sampling sites. Numbers indicate *p* values from nonparametric Wilcoxon or Kruskal-Wallis tests. (b-d) Principal coordinate analysis (PCoA) plots based on Bray-Curtis dissimilarities of (b) all samples, (c) each sampling site, and (d) each compartment. The relative contribution and significance of different factors to community dissimilarity were assessed by PERMANOVA. ‘S’ denotes sampling sites, ‘C’ denotes compartments, and ‘SS’ denotes host species identity. Numbers above the figures (b-d) represent *R^2^* and *p* values from PERANOVA, with *p* < 0.05 highlighted in red. ‘Phyllo’ indicates phyllosphere, and ‘Endo’ indicates endosphere.

**Figure S2:** Effects of sampling site, compartment, host species identity and their interactions on the relative abundances of the top 10 bacterial classes, as assessed using GLM. F-value is indicated by the size of circle and statistical significance is indicated by asterisks (* *p* < 0.05, ** *p* < 0.01, and *** *p* < 0.001).

**Figure S3:** Mean community composition of the top 10 bacterial classes in different host species, compartments, and sampling sites.

**Figure S4:** Pearson correlations between soil properties and the relative abundances of the top 10 bacterial classes in (a) CBL and (b) GS. Positive and negative correlations are shown in purple and green colors, respectively. Statistical significance is indicated by asterisks (* *p* < 0.05, ** *p* < 0.01, and *** *p* < 0.001).

**Figure S5:** Hierarchical clustering based on Bray-Curtis dissimilarity in different compartments based on bacterial community (a, b, c) and predicted functional profiles (d, e, f).

**Figure S6:** Numbers and taxonomic composition of ASVs more abundant in phyllosphere communities relative to soil bacterial communities at each sampling site. (a) Numbers of differentially abundant ASVs in phyllosphere relative to soil in CBL and GS. Red and blue dots represent the more and less abundant ASVs, respectively, with counts indicated. (b) Relative abundance of and taxonomic composition of the top 10 genera based on ASVs more abundant in CBL and GS.

**Figure S7:** Significantly differences in KEGG level two metabolic pathways of differentially abundant ASVs between phyllosphere and soil bacterial communities in (a) CBL and (b) GS, as assessed using Welch’s t-test.

**Figure S1:** Bacterial alpha diversity (a) and functional structure (b-d) across host species identity, compartments and sampling sites. (a) Shannon and Simpson in different host species, compartments and sampling sites. Numbers indicate *p* values from nonparametric Wilcoxon or Kruskal-Wallis tests. (b-d) Principal coordinate analysis (PCoA) plots based on Bray-Curtis dissimilarities of (b) all samples, (c) each sampling site, and (d) each compartment. The relative contribution and significance of different factors to community dissimilarity were assessed by PERMANOVA. ‘S’ denotes sampling sites, ‘C’ denotes compartments, and ‘SS’ denotes host species identity. Numbers above the figures (b-d) represent *R^2^* and *p* values from PERANOVA, with *p* < 0.05 highlighted in red. ‘Phyllo’ indicates phyllosphere, and ‘Endo’ indicates endosphere.

**Figure S2:** Effects of sampling site, compartment, host species identity and their interactions on the relative abundances of the top 10 bacterial classes, as assessed using GLM. F-values are indicated by circle size and the numbers at the bottom. Statistical significance is indicated by asterisks (* *p* < 0.05, ** *p* < 0.01, and *** *p* < 0.001).

**Figure S3:** Mean community composition of the top 10 bacterial classes in different host species, compartments, and sampling sites.

**Figure S4:** Pearson correlations between soil properties and the relative abundances of the top 10 bacterial classes in (a) CBL and (b) GS. Positive and negative correlations are shown in purple and green colors, respectively. Statistical significance is indicated by asterisks (* *p* < 0.05, ** *p* < 0.01, and *** *p* < 0.001).

**Figure S5:** Hierarchical clustering based on Bray-Curtis dissimilarity in different compartments based on bacterial community (a, b, c) and predicted functional profiles (d, e, f).

**Figure S6:** Numbers and taxonomic composition of ASVs more abundant in phyllosphere communities relative to soil bacterial communities at each sampling site. (a) Numbers of differentially abundant ASVs in phyllosphere relative to soil in CBL and GS. Red and blue dots represent the more and less abundant ASVs, respectively, with counts indicated. (b) Relative abundance of and taxonomic composition of the top 10 genera based on ASVs more abundant in CBL and GS.

**Figure S7:** Significantly differences in KEGG level two metabolic pathways of differentially abundant ASVs between phyllosphere and soil bacterial communities in (a) CBL and (b) GS, as assessed using Welch’s t-test.
